# A model of the pre-assessment learning effects of assessment is operational in an undergraduate clinical context

**DOI:** 10.1186/1472-6920-12-9

**Published:** 2012-03-16

**Authors:** Francois J Cilliers, Lambert WT Schuwirth, Cees PM van der Vleuten

**Affiliations:** 1 Centre for Teaching and Learning, Stellenbosch University, Private Bag X1, Matieland 7602, South Africa; 2 Health Professional Education, Flinders Innovations in Clinical Education, School of Medicine, Flinders University, GPO Box 2100, Adelaide, SA 5001, Australia; 3 Department of Educational Development and Research, University of Maastricht, Universiteitssingel 60, 6229 ER Maastricht, The Netherlands

## Abstract

**Background:**

No validated model exists to explain the learning effects of assessment, a problem when designing and researching assessment for learning. We recently developed a model explaining the pre-assessment learning effects of summative assessment in a theory teaching context. The challenge now is to validate this model. The purpose of this study was to explore whether the model was operational in a clinical context as a first step in this process.

**Methods:**

Given the complexity of the model, we adopted a qualitative approach. Data from in-depth interviews with eighteen medical students were subject to content analysis. We utilised a code book developed previously using grounded theory. During analysis, we remained alert to data that might not conform to the coding framework and open to the possibility of deploying inductive coding. Ethical clearance and informed consent were obtained.

**Results:**

The three components of the model i.e., assessment factors, mechanism factors and learning effects were all evident in the clinical context. Associations between these components could all be explained by the model. Interaction with preceptors was identified as a new subcomponent of assessment factors. The model could explain the interrelationships of the three facets of this subcomponent i.e., regular accountability, personal consequences and emotional valence of the learning environment, with previously described components of the model.

**Conclusions:**

The model could be utilized to analyse and explain observations in an assessment context different to that from which it was derived. In the clinical setting, the (negative) influence of preceptors on student learning was particularly prominent. In this setting, learning effects resulted not only from the high-stakes nature of summative assessment but also from personal stakes, e.g. for esteem and agency. The results suggest that to influence student learning, consequences should accrue from assessment that are immediate, concrete and substantial. The model could have utility as a planning or diagnostic tool in practice and research settings.

## Background

Even though the belief that assessment influences student learning is widely proclaimed, attempts in field settings to influence learning in desirable ways using assessment have not been very successful e.g., [1]. One reason may be that even thoughtfully conceived attempts are not informed by a sufficiently theoretically grounded understanding of how assessment influences learning.

While there is much literature relating assessment and learning, there is currently no satisfactory theory or model offering support to the "assessment for learning" endeavour. Calls have been made for a greater role for theory in researching assessment [2] and in understanding what interventions work under which conditions [3]. There has only recently been an attempt to formalize existing knowledge by classifying the learning effects of assessment [4]. A distinction is drawn between pre-, pure and post-assessment learning effects that respectively impact learning before (e.g., study behaviour), during (e.g., portfolios, testing effect) and after (e.g., feedback) assessment.

A validated model explaining (rather than describing) how assessment influences learning could benefit the design of, and research into, assessment for learning. Self-regulation theory has been invoked to explain the effects of assessment [5-8]. Other empirical work resulted in the proposal of a "grade point average perspective" [9], while a synthesis of literature resulted in a model explaining students' study strategies when preparing for classroom tests [10]. None of these models or frameworks have been further validated, however.

**Figure 1:**
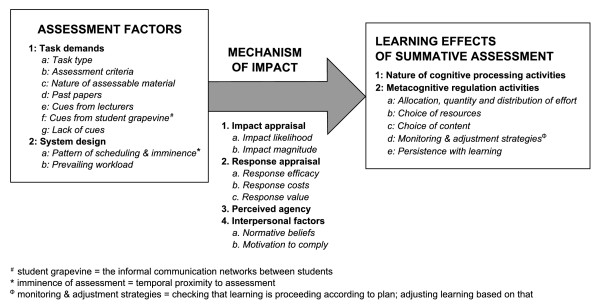
**A model of the pre-assessment learning effects of summative assessment **[11].

Although not typically designed with learning aforethought, summative assessment strongly influences learning. We recently proposed a model explaining the pre-assessment learning effects of summative assessment [11]. According to the model (Figure 1), task demands and system design influence the quality and regulation of learning. These effects are mediated by a mechanism that involves impact appraisal, response appraisal, perceived agency and interpersonal factors. Thus, when contemplating an upcoming assessment event, students may consider the likelihood that assessment will impact them (positively or negatively) and what the magnitude of that impact is likely to be. They may consider the efficacy of any given learning response in bringing about a desired outcome, the costs of that learning response and how the desired or likely outcome relates to their values. Their perceptions of their ability to bring about a particular outcome may also influence their learning, as may their perceptions of the opinions of referents like lecturers and fellow students and their motivation to comply with those perceptions.

The relationship between assessment factors, mechanism factors and learning effects is not a simple one-to-one-to-one relationship. In any given assessment context for any given student, one or more assessment factors will influence one or more aspects of learning, acting via one or more facets of the mechanism. Different students can react in different ways to any given assessment event, depending on what factors in their mosaic of academic and personal motivation enjoy prominence at that time. Equally, any one student can react in different ways to different assessment events, as factors enjoying prominence in their motivational mosaic wax and wane. See Additional File 1: Additional material_Illustrative quotes.pdf for more extensive information on the model.

Whilst this model is grounded in empirical data, it too has yet to be validated. Validation is necessary before any model can meaningfully inform the design of, or research into, the learning effects of assessment. The question now is whether the model can be shown to be valid outside of the immediate context in which it was derived and how to approach this.

To explore this, a clinical setting i.e., a different educational context to that in which the model was derived, held appeal. Furthermore, surprisingly little has been written about the impact of performance assessment in authentic field settings on student learning [12-14]. When contemplating the use of assessment to influence learning in desirable ways, this lack is problematic for fields like the health sciences where performance assessment can comprise a substantial component of assessment.

A qualitative rather than a quantitative approach also seemed appropriate. There are too many variables and relationships to attempt validation using traditional quantitative means. Maxwell [15,16] argues that qualitative research is well suited to taking account of the integral role of context and mental processes in causal processes and understanding. He further argues that prerequisites for the use of experiments in service of understanding causality include well-developed theory that allows interpretation of the results and a manipulable, fairly simple process free from "temporal and contextual variability" [16]. Given the as yet tentative nature of the model, its complexity and the temporal and contextual variability of the relationships in the model, we opted for a qualitative approach at this stage in the development of the model.

Our research question was whether the model could be used to explain observations about the learning effects of assessment in a different context to that in which the model was derived. To do this, we undertook a qualitative study based on in-depth interviews with senior medical students about the impact of assessment on their learning in a clinical setting.

## Methods

### Context

Respondents were South African medical students. Most students at this medical school enrolled directly after secondary school. From semesters four to nine of the six-year curriculum that they followed, students spent alternating four-week periods on clinical theory and clinical practice modules. Clinical theory modules entailed full-time classroom-based instruction on various aspects of clinical medicine. Most of these modules were system-based and multidisciplinary. Much of this instruction comprised lectures; project work and tutorials were less commonly used. Clinical practice modules entailed full-time instruction in clinical settings. Most of these modules were discipline-based. In most clinical practice modules, students worked from day-to-day with preceptors, often registrars. Interaction with consultants tended to be less frequent, sometimes once or twice a week during ward rounds and tutorials.

Students received study guides for each module that spelled out module outcomes and details of assessment. Each module was assessed summatively. Adequate performance in assessment in clinical theory modules gained students access to an end-of-year examination in that module. Students had to pass that examination to progress to the next year of study. Having negotiated assessment in a clinical theory module, typically on the last Friday of that module, students then started with their next clinical practice module on the following Monday. During semesters four to nine, there were no end-of-year examinations for clinical practice modules. Students had to pass assessment in clinical practice modules at the end of each module to progress to the next year of study. Assessment in the clinical practice setting usually comprised a continuous and an exit element. Continuous assessment varied. Students typically had to account for their learning on a daily basis by presenting to preceptors. On some modules, a "ward mark" was awarded based on criteria like punctuality, enthusiasm etc. This mark was usually awarded by the consultant, rather than the registrar, however. Some modules utilized case studies or project work for assessment. Exit assessment at the end of a module often entailed an OSCE, an oral or a clinical case. Having completed a clinical practice module, students would start the next clinical theory module on the following Monday.

### Data collection and analysis

Data was collected by conducting in-depth, unstructured, face-to-face interviews with individual medical students. Respondents determined the venue for and language (Afrikaans or English) in which interviews were conducted. Each interview lasted about 90 min, was audio-recorded and transcribed in full. Each respondent was interviewed once. All interviews were conducted by the same author, an educational adviser fluent in English and Afrikaans, with medical and educational qualifications and training in qualitative interviewing. He worked in the faculty but had little day-to-day interaction with students.

Interviews first explored how respondents learned and how they had been assessed across the course of their studies and then how they adapted their learning in response to assessment. The influence of assessment on learning in a theory teaching context was explored first, then that in a clinical teaching context. Interviews were not structured beyond addressing these three issues in these two contexts. Furthermore, in keeping with the grounded theory design of the study, later interviews were informed and influenced by preliminary data analysis of earlier interviews.

Respondents were asked throughout to provide concrete examples to illustrate how assessment influenced their learning. The influence of assessment on learning was thus explored in various and different contexts and across time, although data was collected at one time point. The dataset comprised over 700 pages of transcriptions.

Previously, an inductive analysis of the dataset was undertaken, utilizing the principles of grounded theory (for details, see [11,17]). This initial analysis drew only on those portions of the interviews relating to assessment in theoretical modules and yielded the model described earlier (Figure 1). For the current study, the dataset was analyzed utilizing the code book developed during the previous phase of analysis, but now focusing on only those portions of the interviews relating to assessment in clinical practice modules. This data had not been included in the initial analysis and was thus being analysed for the first time for this study.

Atlas.ti was used to facilitate this process. We remained alert throughout analysis to data that might not conform to the coding framework and open to deploying inductive coding as needed. Initial analysis was undertaken by one investigator. Results were subsequently refined and finalized during repeated discussions between all three team members.

Respondents had been recruited by addressing and then emailing the fourth (n = 141) and fifth (N = 143) year classes, inviting volunteers to participate in the study. Thirty-two students volunteered for interviews. Interviews were conducted with the first 18 students who volunteered (Table 1). The remaining volunteers were thanked but not interviewed. This decision was taken when no new data emerged after interview 14, despite later interviews being individualized to explore constructs emerging from preliminary analysis, which analysis was initiated whilst interviews were still being undertaken. During initial analysis, no new constructs emerged after interview 12.

**Table 1:** Summary of respondent characteristics based on year of study, gender and academic performance across all 6 years of study

		Average mark		
**Year of study**	**Gender**	**< 70%**	**70-79%**	**≥ 80%**

4	F	Resp13		
	
	M		Resp7	
			Resp16	

5	F	Resp6	Resp2	Resp4
		Resp12*	Resp11*	Resp8
		Resp15*		Resp9
		Resp17		Resp18
	
	M	Resp3	Resp5	Resp1
			Resp14	Resp10

*: Respondent failed one/more modules during their studies

An institutional research ethics board granted ethical approval for the study. Informed consent was obtained from respondents for participation in the study and later access to their study records.

## Results

All major components of the model were evident in the clinical context. Relationships between components could all be related to the model. These findings will be summarized briefly below.

One new source factor was found to be operating as a subcomponent of task demands i.e., interaction with preceptors. The model was utilized as a lens to scrutinize the interrelationships of this new subcomponent with previously described components. These results will be reported in greater detail to illustrate how the model operated in the clinical context.

### An overview of findings

#### Assessment factors and learning effects

Task demands and system design were discernible as assessment factors, the nature of cognitive processing activities and metacognitive regulation activities, as learning effects of assessment. The relationships between assessment factors and learning effects are summarized in Table 2 and compared to findings emanating from theoretical modules.

**Table 2:** Comparison of how assessment factors and pre-assessment learning effects of assessment are linked in theoretical and clinical practice modules

		LEARNING EFFECTS OF ASSESSMENT											
				**Metacognitive regulation activities**									
**ASSESSMENT FACTORS**		**Nature of cognitive processing activities**											
				**Allocation, quantity & distribution of effort**		**Choice of resources**		**Choice of content**		**Monitoring and adjustment strategies**		**Persistence with learning**	

**Task demands**	Task type	T	C	T	C	T	C	T	C	T	C		
	
	Assessment criteria	T	C	T	C	T	C						
	
	Nature of assessable material	T		T		T	C	T	C				
	
	Interaction with preceptors		C		C		C		C				
	
	Past papers	T				T		T		T			
	
	Cues from lecturers	T	C	T	C	T	C	T	C				
	
	Cues from student grapevine	T		T	C	T	C	T	C	T			
	
	Lack of cues					T		T	C			T	

**System design**	Pattern of scheduling &imminence	T	C	T	C	T	C	T	C	T		T	C
	
	Prevailing workload	T	C	T	C	T	C	T	C	T		T	

This table illustrates the associations between assessment factors (row headings) and learning effects (column headings) for the assessment system as a whole. Where an association between an assessment factor and a learning effect was identified during data analysis, the intersecting cell in the table has been labelled. The learning effects of assessment are shown for both theoretical modules (cells labelled T) and for clinical practice modules (cells labelled C). In the clinical setting, one new factor was found to be at play as a subcomponent of task demands i.e., interaction with preceptors. One subcomponent of task demands i.e., past papers, was not at play in the clinical setting. Other than that, each assessment factor and each learning effect was involved in an association with at least one subcomponent from the other group in both settings

There were a few notable differences between findings from the two settings. In keeping with the nature of clinical assessment, past papers did not feature as a subcomponent of task demands in the clinical context. Fewer sources of impact influenced monitoring and adjustment strategies or persistence with learning in the clinical context. One new assessment factor was found to be operating as a subcomponent of task demands i.e., interaction with preceptors.

#### Mechanism

All four components of the mechanism of impact were discernible in the clinical context. Examples will be given in the next section.

### A new subcomponent illustrates the operation of the model

Three facets of respondents' interaction with preceptors influenced their learning via the components of the mechanism. These were regular accountability to preceptors, personal consequences that could accrue from these interactions and the emotional valence of the learning environment. Response appraisal was largely geared towards addressing these issues, rather than achieving an academic result. The operation of the model will be described for each of these facets rather than for the subcomponent "interaction with preceptors" as a whole. Model constructs will be highlighted in a bold font. The type of model component will be indicated as follows: AF = assessment factor; MF = mechanism factor; LE = learning effect.

#### Regular accountability

Respondents allocated daily effort to learning (LE: **allocation of effort**) from the start (LE: **distribution of effort**) of a clinical practice module because consequences (MF: **impact likelihood**) were constantly imminent (AF: **imminence**), as they presented information on and had to answer questions about patients.

[Quote 1] a large class is where it's one doctor for 180... [the chances] that you will now be asked are very small... whereas in clinical, you cannot really hide... you must say something (Resp14)

Respondents' learning was influenced more by the immediate and concrete consequences (AF: **imminence**; MF: **impact likelihood**) that accrued during regular exposure on ward rounds (AF: **pattern of scheduling**) than by more distant academic consequences e.g., impact on marks or progression, or ultimate ability to deliver good clinical care. This elicited more regular and evenly distributed "snack-learning" (LE: **distribution of effort**; LE: **quantity of effort**) by respondents than did the more periodic assessments on theoretical modules that induced periodic "binge-learning".

[Quote 2] "[a clinical rotation] is a bit different to [a theoretical module] because you basically have a round every day or an academic round with questions. So then you are tested every day, so you will learn a bit more, more regularly" (Resp13)

#### Personal consequences

Appraisal of **impact magnitude **(MF) also influenced respondents' learning, given that not being able to provide satisfactory inputs when asked on ward rounds could result in profoundly negative personal consequences (see also Quote 7).

[Quote 3] the students talk about how you "bleed" on a ward round if you get to a place where your knowledge runs out and you just get chewed out for that (Resp7)

Appraisal of **response efficacy **(MF) in the clinical context often related more to addressing self goals e.g., preserving esteem, through avoidance of negative intrapersonal consequences (i.e., avoidance goals) than to addressing assessment-related performance goals (i.e., approach goals). It was more a case of "If I do this, will it keep me 'safe' on the ward round tomorrow" than of "If I do this, will it help me pass (well)". The stakes are personal rather than assessment related, but nonetheless high.

[Quote 4] some consultants are more - how can I say, more pushy than others. ... you tend to prepare and prepare and prepare because you are preparing more out of fear of being humiliated there than out of the understanding as such. (Resp12)

The prominence of personal consequences was not due to the lack of other sources of influence. Students had to achieve satisfactory marks for continuous and for exit assessment to pass the module and the year.

#### Emotional valence

The emotional valence of the learning environment was closely linked to, but distinct from, personal consequences and was generated by preceptors. Two extremes were discernable from characterizations by respondents of preceptors, called here "tyrants" and "teddy bears".

[Quote 5] You just get your two extremes, the one that will make you break out in fear and trembling, so you will... you will just learn because, yes, you don't want to continue to live in fear. And then you get those that... that are very nice and that explain everything nicely to you... that doctor, you don't want to disappoint either. ... for that doctor, I will almost go even further... go out to learn, to bring back information and so on, to participate. (Resp14)

In the quote below, the respondent describes positive (bold) and negative (italics) learning environments created by different preceptors and her respective learning responses (underlined):

[Quote 6] [names consultant from one discipline] is a **sweetie pie**. ... **you have to know your work. ... you have to go and read up**, but you can do it more calmly. You know, if you don't know something, **you can tell him you don't know**. You can remember it, but **you can check in your notes** and you can say "oh, this, you know, this is this". It's a **much more relaxed atmosphere **that you're going to read the stuff in. ... [names consultant from another discipline]... then *you go and sit and you swot until you can remember that stuff. And every last thing ... you can't leave anything out*. ... he asks the most impossible, weirdest stuff... so *you try to read up everything that you can*. [You do it] because *he is very scary*. He *rebukes you *such that *you feel smaller than a snail*. ... So you try as far as possible not to place yourself in that situation. ... [But] it's a crammed situation... *one's anxiety levels are so high, you literally sit and force that stuff, that you can almost remember a page just so*. But *as soon as that presentation is finished, everything flies out of your mind*, but if I've read for [names 1st consultant again], **the stuff stays with me** and **I'll go and read about the stuff again in the evening**, but with [names 2nd consultant again] I'll never... *I'll just make sure I know the next day's work. I won't still be interested in doing other stuff*. (Resp6)

For preceptors characterized as tyrants, learning was a self-defence mechanism, driven by a performance avoid goal orientation. Appraisal of **impact likelihood **(MF) and **impact magnitude **(MF) resulted in effort being allocated (LE: **allocation of effort**) with a view to avoiding censure on ward rounds (see also Quote 4). Appraisal of **response efficacy **(MF) led respondents to conclude that they had to come to a ward round knowing "everything" (i.e., everything necessary to "stay safe" on that ward round), so they allocated extra effort (LE: **quantity of effort**) to learning. They selected content to learn (LE: **choice of content**) based on what the **student grapevine **(AF) indicated was what the preceptor wanted to hear. Avoiding the wrath of the consultant (MF: **impact magnitude**) gave short-term utility (MF: **response value**) to learning even material that was perceived as irrelevant to respondents' longer-term goals of becoming good generalist clinicians. Material was committed to short-term memory, rather than being incorporated into mental models (LE: **nature of cognitive processing activities**), and then forgotten with alacrity once the ward round was over. Furthermore, once the ward round was over, the motivation to learn dissipated, and no further reinforcement (LE: **distribution of effort**; **monitoring & adjustment**) took place. Respondents would not ask questions for fear of being ridiculed or attacked (MF: **impact magnitude**) for not knowing something they should already know.

[Quote 7] I feel more at liberty to ask [registrars], to ask easy questions, than I would asking a consultant. ... Because [consultants] maybe expect that I should know it. ... [I don't ask consultants] because I am scared they scold or get abusive ... and yes, I try and avoid that. While ... a [registrar] will explain to you quite nicely. [The consultant] will say to you "That class, did you not... do you not remember that class I gave, so and so much time ago", while the [registrars] will explain again nicely. (Resp13)

For preceptors characterized as teddy bears, appraisal of **response value **(MF) featured more prominently. **Effort was allocated **(LE) so as to be seen to be "doing your bit". Respondents were more likely to learn material of general relevance and of interest to themselves (LE: **choice of content**).

[Quote 8] then you get other... like I think of [names consultant]. I was with him for one week in third year, but I enjoyed that so much, because he... he made us think and he explains stuff and he just made us so interested. But you weren't... it wasn't like you really were reading up stuff out of fear, because you... if you didn't know something it wasn't like you were any less of a person. And that's much more pleasant. ... if you're relaxed, you feel free to ask questions and you actually enjoy the module then you... you know, you find the stuff interesting. (Resp4)

Learning was driven by a mastery approach goal orientation, with self-regulated reinforcement of learning taking place after the ward round (LE: **distribution of effort**; **monitoring & adjustment**). Furthermore, respondents perceived teddy bears to be resources.

They felt that if they did not know something, they could ask teddy bears on ward rounds (LE: **choice of resources**) and learn from them. This is not to say that teddy bears did not have high expectations. They did (see Quote 6). The learning environment they created was very different, however.

## Discussion

The purpose of this study was to explore whether the model describing the pre-assessment learning effects of assessment had explanatory potential in a clinical setting. In this study, the model could be utilized to analyze and explain observations generally in an assessment context different to that in which it was originally derived, albeit for the same group of respondents as were involved in the development of the model initially. One subcomponent of task demands, past papers, logically played no role in this context and one new context-specific subcomponent, interaction with preceptors, was identified. The model proved useful to explain the operation of this new subcomponent. To the best of our knowledge, no other model of the learning effects of assessment has been validated to any extent. Thus, while this study has various limitations (addressed below), we nonetheless believe it is an important - if modest - first step in what is typically a substantial and ongoing process [18] of model validation. We also believe that these results have ecological validity [19], being derived from the lived experiences of respondents with no experimental manipulation.

Our results further demonstrate that the model is operational outside of a strictly summative assessment context. Summative assessment is not the key construct driving learning here, yet the model offers an explanation of what is happening. Conditions here were clearly "consequential" [6] even though consequences for performance on assessment were not the primary consideration. This model is thus perhaps better conceptualized as one of the pre-assessment learning effects of consequential assessment.

The role of preceptors was an unexpected finding. Sadly, our respondents' characterization of "tyrant" preceptors supports other similar findings. Adverse interactions with preceptors have variously been characterized as an issue of style [20] or educator professionalism [21] and as involving disrespectful interactions, belittlement, humiliation, bullying, harassment and abuse [22-27]. Our findings add to a fairly limited literature describing the consequences of such adverse interactions [22-24,28] and, further, provide an explanation of why students responded in the way that they did in our setting.

Our results yield some guidance for practitioners about the design of "assessment for learning". A supportive, low-risk learning environment characterized by high expectations and limited personal consequences resulted in deeper cognitive processing activities. In contrast, a negative, high-risk environment characterized by high expectations and negative personal consequences resulted in surface cognitive processing strategies and low engagement e.g., no question asking. To influence learning, assessment should be consequential and yield consequences that are concrete and substantial rather than abstract and trivial. Making assessment summative is one way of making assessment consequential. However, influencing personal stakes e.g., for esteem or agency and influencing emotion can also elicit (positive and negative) learning responses. Imminence of consequences is also important, with immediate consequences having a stronger influence than more deferred consequences.

One question this work raises is how to induce even distribution of learning effort with assessment without burning students out with unrelenting demands. Considered together with how respondents reacted to assessment in theoretical modules [11], it seems evident that regular accountability in clinical practice modules played a central role in determining students' overall learning pattern and had a negative knock-on effect on learning in subsequent theoretical modules. In theoretical modules, respondents described taking time off at the start of a module to "catch up" with the rest of their lives. In clinical practice modules, there was no indication that learning could be side-lined like that. Although not vocalized in interviews, it is tempting to speculate that the demands of the clinical setting were so pervasive and continuous that respondents had little opportunity to take time off to spend on non-academic aspects of their lives; that they were exhausted when they started the next theoretical module, and, being able to do so, took time off from their studies. The costs of doing so were less immediate in theoretical than in clinical practice modules, as was the likelihood of impact. Respondents did realize, though, that there was a deferred impact in the form of a higher workload in the run-up to assessment in theoretical modules. This "deferred" cost was deemed worthwhile, however, for the opportunity to devote time and attention to other aspects of their lives.

A potential limitation to this work is that it was undertaken with one group of undergraduate medical students in South Africa. Indeed, the data upon which this analysis is based were collected from the same group of respondents at the same time as was the data from which the model was derived originally. This analysis was conducted utilising a subset of data from that extensive dataset, though, data that had not previously been analysed. Ultimately, this is not considered a drawback as the purpose of the study was not to yield generalizable results but to explore the explanatory potential of the model. It could be argued that using a qualitative approach as a first step in the validation of the model is also a limitation. Again, given that explanation rather than prediction [18,29] was a goal, this is not considered a drawback. In fact, this approach revealed a new construct at play i.e., interaction with preceptors, something that would not have been evident had a variance theory approach been adopted at this early stage of the validation of the model.

Future research should establish whether the model meets other criteria for validity such as have been proposed in the literature [18]. Stronger evidence to support or falsify propositions needs be sought using quantitative methods; so too stronger evidence for explanatory power. Future exploration of generalizability should include the determination of whether the model is operational in other settings e.g., other universities and other disciplines. It would also be interesting to explore whether this model is operational in low-stakes assessment settings and in postgraduate settings.

## Conclusion

The model could have utility as a planning tool, to help prospectively plan an assessment system, or as a diagnostic tool, to help evaluate and enhance the learning effects of assessment in existing systems. However, it will be necessary to develop simplified instruments to utilize for this purpose.

## Competing interests

The authors declare that they have no competing interests.

## Authors' contributions

FJC contributed to the study conception and design, and data collection, analysis and interpretation. He drafted the manuscript and revised it in accordance with suggestions from the other authors. LWTS contributed to the study conception and design, data interpretation, and critical revision of the manuscript for important intellectual content. CPMvdV contributed to the study conception and design, data interpretation, and critical revision of the manuscript for important intellectual content. All authors read and approved the final manuscript.

## Authors' information

FJC is a Deputy Director at the Centre for Teaching and Learning at Stellenbosch University in South Africa and has an MBChB and an MPhil(Higher Education). He is also a FAIMER Fellow. LWTS is Professor of Medical Education with Flinders Innovations in Clinical Education, Health Professional Education at Flinders University in Australia and has an MD and a PhD. CPMvdV has a PhD and is Chair of the Department of Educational Development and Research, Scientific Director School of Health Professions Education, Maastricht University, The Netherlands, Honorary Professor at the University of Copenhagen, Copenhagen, Denmark, King Saud University, Riyadh, Saudi Arabia and Radboud University, Nijmegen, The Netherlands.

## Pre-publication history

The pre-publication history for this paper can be accessed here:

http://www.biomedcentral.com/1472-6920/12/9/prepub

## Supplementary Material

Additional file 1 **Additional material_Illustrative quotes**. More extensive information on the model is provided in the additional file Additional material_Illustrative quotes.pdf. Descriptions of constructs are provided as are illustrative quotes for each. Click here for file
