# Supplementary material for: A model of the pre-assessment learning effects of assessment is operational in an undergraduate clinical context
Source: BMC Med Educ. 2012 Mar 16;12:9. doi: 10.1186/1472-6920-12-9 (PMC3375194; doi:10.1186/1472-6920-12-9)
Supplement: Additional file 1 — Additional material_Illustrative quotes. More extensive information on the model is provided in the additional file Additional material_Illustrative quotes.pdf. Descriptions of constructs are provided as are illustrative quotes for each. [file 1472-6920-12-9-S1.PDF]

ADDITIONAL FILE: ILLUSTRATIVE QUOTES

SOME QUOTES TO ILLUSTRATE A MODEL OF THE PRE-ASSESSMENT LEARNING EFFECTS OF CONSEQUENTIAL ASSESSMENT

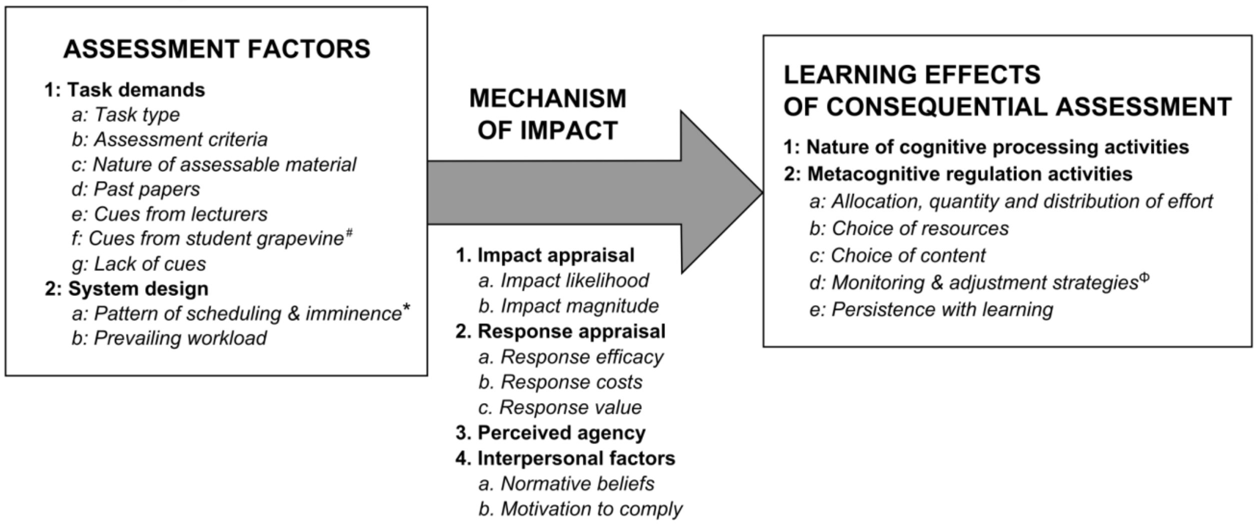

# student grapevine = the informal communication networks between students
* imminence of assessment = temporal proximity to assessment
$^{\Phi}$ monitoring & adjustment strategies = checking that learning is proceeding according to plan; adjusting learning based on that

The derivation of this model has been described elsewhere [1, 2]. This document is intended to equip a reader encountering the model for the first time with sufficient background to understand the model's constructs and their interrelationships. The model comprises three major components i.e., assessment factors, mechanism factors and learning effects.

The interplay between these can be visualized as a three-dimensional matrix. In different scenarios, different assessment factors and mechanism factors combine in varying intensities to yield different sets of learning effects. Not all factors are active in all students at any given time or, indeed, the same student at different times. Furthermore, different sets of interactions could be at play for different students in the same assessment context and for the same student in different assessment contexts.

The relationship between assessment factors and learning effects was mutually multiplex i.e., any given assessment factor influenced various learning effects and any given learning effect was influenced by various assessment factors.

The following quote illustrates the interplay of factors.

> [Resp1] … in the earlier years… say first, second and third year you were thinking more along the lines of… I must pass… I must get through this exam. … But now, when I started hitting last year and this year you start thinking. I'm actually going to be a doctor. It's no good to me using a way to memorize these facts when I'm not going to be able to use it practically in my job one day. … I've started trying to change the way I study… … the way I approach a module is trying to maybe within the first two or three weeks of a four week block, to understand… to understand the concepts more and to sit with the concepts and try to work the concepts out rather than just memorizing. And then it's unavoidable when it comes to the last week, last week and a half of a block … You just try and cram - try and get as many of those facts into your head just that you can pass the exam and it involves… sadly it involves very little understanding because when they come to the test, when they come to the exam, they're not testing your understanding of the concept. They're testing "can you recall ten facts in this way?" … And yes you know that I'm gonna be asked to recall five facts. So then you just learn

five facts rather than trying to understand the core concepts.

As assessment becomes more **imminent** ("*when it comes to the last week, last week and a half of a block*"), **impact likelihood** ("*it's unavoidable*") and **impact magnitude** ("*just that you can pass the exam*") are considered, along with **response value** (success in assessment increasing, patient care decreasing in value as assessment looms). These factors, together with **task type** ("*they're not testing your understanding of the concept. They're testing 'can you recall ten facts in this way?'*") and **response efficacy** ("*You just try and cram - try and get as many of those facts into your head just that you can pass the exam*") considerations, generate an impact on the **nature of cognitive processing activities (CPA)** ("*So then you just learn five facts rather than trying to understand the core concepts*").

Adapted from [2]

What follows is a description of each construct and a quote or two, some of which have been used in previous descriptions of the model. These quotes are not meant to be exhaustive but rather illustrative. Please refer to the papers originally describing the model [1, 2] for more complete descriptions. Any given quote may illustrate more than one construct and may thus be repeated. As you read these quotes, bear in mind who the respondents are that said these things, especially noting their average score over six years of medical school:

		**Average score over six years of medical school**		
**Year of study**	**Gender**	<70%	70-79%	≥80%
4	F	Resp13		
	M		Resp7<br>Resp16	
5	F	Resp6<br>Resp12*<br>Resp15*<br>Resp17	Resp2<br>Resp11*	Resp4<br>Resp8<br>Resp9<br>Resp18
	M	Resp3	Resp5<br>Resp14	Resp1<br>Resp10

*: *Respondent failed one/more modules during their studies*

A. ASSESSMENT FACTORS

A1 TASK DEMANDS

A1a. task type:

students infer task demands directly from the type of assessment task to be used during an assessment event or indirectly based on their perceptions of the complexity of the cognitive challenge posed

> **(Resp4)** If I go back to to second year [names module]. *There was those true false questions ... You didn't study like in facts in the way that you would if you had to give a paragraph. You'd study it to understand it, because that's basically the true false questions were testing your understanding*. ... if it's going to be multiple choice questions ... this and this is a complication of that, you only need to recognize it. You don't have to think it out yourself. ... if you've seen it before and you understand how it makes sense as a complication, you're not going to study it

> **(Resp6)** at the end of the day, *you leave pretty important stuff* [referring to pathophysiology] *out for now to learn ridiculous lists of thingeys*. … You know you must get these little lists in your head, but at the end of the day, your insight in… in the whole story is… left out. Now if you know the pathophysiology, you can figure out

almost anything. So, you leave out pretty important things because you don't have time to swot them. … *[I learn lists] because that's what they ask. It's pretty much what they ask. You know then they will say, name five causes of this for me. Or list 10 differential diagnoses of this. You have to give lists.*

A1b. assessment criteria:

the criteria students perceive to be used to grade assessment products and how they perceive those criteria to be applied e.g. strictly or loosely

(Resp1) in the theory modules, you know that they are marking according to a memo … very often even if you are correct, because it's not on the memo, they do not mark it correct. I've had that experience a number of times. So if I now write in my test paper … *you're very worried that you're going to be marked incorrectly for [the] sthinking that you are thinking. You'd rather... give it to them in the exact form that they gave it to you*. … *if you had to be examined by, say for example, [names professor* from another module to that referred to above], *he is very for lateral thinking and open minds, so you wouldn't study in the sense of trying to just recall those ten facts. You would more have an opinion on certain topics.*

A1c. nature of assessable material:

how easily material being studied for assessment can be grasped

(Resp10) … [names subject] is not very logical. *You cannot reason it out for yourself ... unless you go and look in super-depth ... at almost ... micro pathological level and ... I have neither the time nor the interest to go and do that*. … I guess it's to a large extent about motivation to really understand that work … *[names subject again] is pretty much for me, you know, I must pass it. That's all I felt about it. It's not something I particularly wanted to do well or wanted to understand.*

(Resp11) it also depends a lot on… on the type of work you are swotting*; because *one thing is such that you can sort of explain it to yourself and another thing is, like, a little list. So then you must rather think of a way to remember that little list most easily.*

* swot: study, especially intensively in preparation for an examination

A1d. past papers:

cues inferred from previous assessment tasks

(Resp7) what I will do is *I will see a question paper, I browse through the whole paper, the whole paper is just five to ten point "listing" questions.* They're not paragraph or insight or case-studies… Or the case studies are actually just again just to get the little lists out of you in another way… And so *I will concentrate on that. If I page through that work and here is some or other description of a thing, I'll maybe skip over it, but you'll stop at a five or ten point list.* Yes, then one just swots like that, you know, that is what will get you through.

(Resp12) … *closer to the time I would sort of have glimpse of a question paper if there's any*. Yes, I think I usually use a question paper more than the actual outcomes. … sometimes the outcomes are a pain to try and understand what exactly they are. And then so *I use the question paper exactly as I should have been using the outcome, to try and understand what is more important.*

A1e. cues from lecturers:

cues inferred directly or indirectly from lecturers

(Resp10) *the lecturers do rather tend to give spots and then I always feel, okay, now I know that question will be asked. I think that it is a stupid question to ask, but I reckon it will be asked. Then I just go and swot that list* … sometimes they are very direct, the spots. You know, what lecturers say to you this is a type of thing that he will ask in the exam and so on. Other times, you know, it sort of comes via the grapevine, you just hear that this is important. … It's also not necessarily always spots. *They will say, I almost want to say negative spots too. This is not important for the test, but it is for the exam*. ... obviously you have the past papers, but I mean we also have… I mean, the way that classes are presented. … *if the lecture goes on with a guy that puts up PowerPoint's that click, click, click, click, click and here comes a bunch of information, the next slide. You know, he's not really going to test your insights*, because he didn't try and explain the concepts to you at all. He just simply

gave you facts. So you can just expect that the paper will be factual.

A1f. cues from student grapevine:
cues obtained from peers

(Resp6) *you talk to your friends a lot.* You know, *you listen and you talk in a group and you say 'what are you going to swot, what are you going to leave?'.* Then someone says to you 'but they heard don't swot that'.

(Resp7) As far as assessment goes, I'll easily go and look at an old question paper or two or so, *tips that other students say*, and based on that, I'll go... go learn, focus on certain things

A1g. lack of cues:
where students could not discern cues about what to expect in assessment

(Resp3) in [names a module] … they asked almost no GP stuff. *They asked us very rare stuff.* What I mean, it's superspecialist stuff that I don't think is relevant to the general GP. … *it almost forces you to learn everything and then one cannot say there's a safety… thingey… and say "That is a spot". Then one just tries to focus on everything.* … Then one already knows psychologically, before a module starts: but now I must pay more attention in class… make more good notes and work harder.

A2. SYSTEM DESIGN

A2a(i). pattern of scheduling:
how assessment tasks are distributed in time

(Resp2) *I think that because we write tests periodically, I swot periodically.* … I think [the block method] has definitely caused me to change from a person who worked continuously to a person who does the minimum for the first three weeks of every month and the maximum in the last week.

(Resp9) sometime you misjudge yourself with the amount of work … Then as you get closer to the end, then you see 'oo, crumbs, I'm not going to get finished'. Then either you work harder, which you just do, or *you decide ... this pack and this pack I'm going to leave out. ... then I know for the exam, okay, I didn't learn that pack ... and that pack and that pack very well and then I will plan my exam in such a way that there is still enough time* for me to go through my old work and… *to summarise the work I didn't learn well for the test well and go through it well*

A2a(ii). imminence:
the temporal proximity of assessment at any given moment in time

(Resp1) depending where you are in the module depends on how many sorts of extra mural activities you get yourself involved with. … at the moment I'm like "you know, I really don't feel like studying". So you find all these excuses in the book. Like "it's soccer tonight. I'll just watch the soccer game". Then after the soccer "I'm tired, I'm gonna go to bed". In bed by half past ten, eleven. But then *when the test approaches, it's like you go into a different mode completely. You just almost become a different person. You know exactly what's expected of you. You know that you have to put in these hours* otherwise you are going to come short and you just adapt yourself… you modify yourself.

(Resp13) [When I sit with a pack of notes] I at least have the textbook next to me too, so if I, say now with one or other disease… I'll just scan quickly, quickly, quickly in the book if there is something more than is in the notes. … the PowerPoint is often just the points, the book is at least a little more a story to really gain some insight as to what it all is… so I will quickly read through that before I read just the PowerPoint just like that. *But that is at the beginning then there is still lots of time, and if there is less time, then I just go through the PowerPoint. ... Yes, here around the second week... about four days before the test, I won't do that any more.*

A2b. prevailing workload:
the workload at any given moment in time

(Resp6) *four weeks for such a bunch of work is very little. Is too little.* You don't do it in a long term… it doesn't go in your long term memory. … *if you have more work, you are going to have to swot more superficially and swot more selectively too*

(Resp10) If there is really a bad lectures or study material that one had, before I [will use journals, the internet or PubMed] … it would have been very nice, but I mean there is plain

and simply not enough time. I know [names module] last year… I mean our notes were unbelievably poor and out textbook was even worse. And I was forced… I looked at what they put up as other resources and then at the end of it all, I pulled a couple of [continuing medical education] articles. And it was very nice. … but *it was the only time that I ever used the internet or such places in my course, yes… ... You just don't have time. It would have been very nice, but I just don't get the time to do it.*

B. MECHANISM FACTORS

B1. IMPACT APPRAISAL

B1a. impact likelihood:
how likely consequences are to accrue

(Resp6) … [names module], it's a massive chunk of work … you must swot so selectively about what you are going to leave out and it's not as though you leave out less important things. *You leave things out that you think they will not ask.* So it's maybe big things or maybe important things that could save a patient's life one day, but *you don't swot it because you have to pass the test now* and that's a problem for me.

B1b. impact magnitude:
what the magnitude of consequences (negative or positive) is likely to be

(Resp2) … initially, I studied more, as sick as it may sound, it was really actually nice to learn new things. Now, it's more so that I know when I go and write exams, it's just for my own peace of mind too. *One learns so that you know you are not going to fail*. That's a big motivation.

(Resp7) … as far as assessment goes, I will easily go and look at an old question paper or two or so, tips that other students give and based on that, I will go… go learn, focus on certain things. And, to my own detriment for the day that clinical comes, skip some things, so then I didn't emphasise those, but when you're in a corner… when you are calmly underway, then you feel "I must just swot, uhm… I want to swot to be a good doctor", but when you are in a corner, then you swot to make one exam, because you know uhm… Yes, the pressure is pretty high some days, because then *you know that your whole year can hinge ... on this one exam. In theory, you can plug your year… so then some days all the good intentions go out the window.*

(Resp8) …we are also assessed along the way with little assignments that we usually have to hand in. And sometimes there will be more than one small test during the module, that also contributes a bit. But *my big problem with that is that it usually counts so little…* that they give you an assignment that's maybe going to keep you busy for three days and you are going to have to devote all your attention to that if you really want to make a decent effort. And then *it takes up so much of your time and then it counts 10%.* And now you've missed three days learning that you could actually have done for the exam that counts 90%. ... *I don't think I learn anything, because I am busy just rushing it off most of the time, if I see that it is not going to count very much. ... in the beginning, you are a bit more committed, but once you start realising that the work that is covered in the assignments won't necessarily be asked again in a test or... then, I must say, I don't put as much effort as I can into it.* I prefer to rather learn for the test at the end. That's more important to me.

B2. RESPONSE APPRAISAL

B2a. response efficacy:
the efficacy of any given learning response in achieving a particular outcome

(Resp10) I can swot lists for tests, but I forget those again. And it… it frustrates me unbelievably much if I don't have insight. … [I swot lists] because that is the only way I… well, *I must get the marks. You know you must pass your course to become a doctor*. Whether you agree with what they asked or not.

(Resp4) I think you tend to stress more earlier on the course … *You know you can get away with more later on, so you maybe start studying a little later. If you have experienced before that, okay, I can actually start at this stage and still be fine, then you… then you tend do it the next time in the same way.*

(Resp1) if the person has a reputation of setting incredibly tough papers or papers which do not correlate very well with papers which have been set in the past… so if you know that this guy is going to set you a paper

with questions which he has never set before and you will not find in previous test papers and which are going to be very difficult questions, *your approach will be a lot more thorough and you won't just skip out in things, saying "He'll never ask that, he'll never ask that"*

B2b. response costs:

the costs of any given learning response; costs can be incurred by responding, and not, to the demands of assessment and may be internal or external to the student

(Resp6) … the whole approach of four weeks for one large subject like [names module], it's a massive chunk of work and then you must swot so selectively about what you are going to leave out and *it's not as though you leave out less important things. You leave things out that you think they will not ask. So it's maybe big things or maybe important things that could save a patient's life one day, but you don't swot it because you have to pass the test now and that's a problem for me.*

(Resp9) *… some nights, I learned until 23:30 and got up 06:00 the next morning. But then… then you're in, like, super-stress mode.* Then you do it of course because you think you are not going to pass. … I think if there is no assessment, you won't learn, because that is basically what it's about in medicine. You have a four week module, for example, and you know the first week that you must get an overview of the work and then *you know when your stress mechanism starts to kick in, you must now start learning and then you start learning. [I stress when] I feel if I didn't go through all the work properly… I won't pass the test.*

(Resp8) …we are also assessed along the way with little assignments that we usually have to hand in. And sometimes there will be more than one small test during the module, that also contributes a bit. *But my big problem with that is that it usually counts so little… that they give you an assignment that's maybe going to keep you busy for three days and you are going to have to devote all your attention to that if you really want to make a decent effort. And then it takes up so much of your time and then it counts 10%. And now you've missed three days learning that you could actually have done for the exam that counts 90%.*

(Resp4) sometimes the modules do that. So it's a four weeks module and you have [a test] two weeks into the modules and then a test at the end. Some of them do that. … I don't like it. Because, obviously *you get to the stage of like, when you feel you've worked hard on your last clinical block and you feel like relaxing, socialising*, then you see you've got a test. The first week of the module: not a good way to start. But I mean it definitely makes you start earlier.

B2c. response value:

the value of any given learning response as measured against the student's personal goals and their conceptions of success and wellness

(Resp8) … in our third year, we could progress [to the next year], so if you got 65, then you didn't need to go and write exams. Now, I cum'd all my theory modules at the end of the day… and so I didn't go and write the exams. And the one… there I go and get 65 on the nose. Then my parents said "are you going to do the exam". Then I said to them "no, I've got 65. I'm going to progress". *I'm not prepared to just, so that it will stand on paper that I cum'd that thing too, now put myself through all that stress of going to learn again and write an exam again. ... to go through the work again another time is, I suppose, always advantageous, but at that time, the pros and cons were just for me… it was just not that important to me.* So, my knowledge would definitely have been better if I went and learned all that work again, but I don't think my mark would necessarily have improved.

B3. PERCEIVED AGENCY

the perception of being able to exert some control over a situation, even in the face of adversity

(Resp8) You know how long it takes you to learn something, when you must start waking up and you know when you are behind. And *you know what your abilities are and how much you must do to…* to be able to get to the test.

(Resp4) you feel less secure if you know you're going to get asked an interpretive question. Whereas you know it's going to be just sort of… like parrot fashion type questions, then you can pretty much study and know you're going to do well. But *if it's*

going to be interpretive questions, you never quite sure exactly what they want.

B4. INTERPERSONAL FACTORS

B4a. normative beliefs:

the beliefs of referents, people (like lecturers and fellow students) whose opinion a student values

B4a(i). normative beliefs – lecturers:

these were typically inferred as opposed to necessarily espoused or enacted beliefs

(Resp2) one gets a reasonable idea in later years what the lecturers think is important. So, if I can, I will swot everything that there is to swot, but *if the time gets a bit too little, then* I take… not chances, but then *I concentrate more on the things that to me are clearly more important*.

(Resp7) *My theory is that a lecturer will not ask me something in an exam that he did not go to the trouble of mentioning in class*.

(Resp12) I use the question papers to see what is more important and to, to test whether I can answer it. … *I believe that, that is my belief, that everything that is important is summarised in these question papers*.

B4a(ii). normative beliefs – students

(Resp7) *I'll talk to guys that are a year or two ahead of me. ... I'll talk to them about how they experienced it*, and they will… *I'll talk to a couple of people, more or less in every block I start*, and then you'll hear, then they say "… wow, they asked this big question in the test, but they said they'll never do that again" or "no, it was easy; look at the papers, they repeated the papers' questions a lot" or they will say "listen here, start really early"

B4b. motivation to comply:

students' motivation to comply with normative beliefs

(Resp7) … as far as assessment goes, I will easily go and look at an old question paper or two or so, tips that other students give and based on that, I will go… go learn, focus on certain things. And, to my own detriment for the day that clinical comes, skip some things, so then I didn't emphasise those, but when you're in a corner… when you are calmly underway, then you feel "I must just swot, uhm… I want to swot to be a good doctor", but *when you are in a corner, then you swot to make one exam, because you know uhm… Yes, the pressure is pretty high some days, because then you know that your whole year can hinge ... on this one exam. In theory, you can plug your year… uhm… so [laughs] then some days all the good intentions go out the window*

C. LEARNING EFFECTS OF ASSESSMENT

C1. QUALITY OF LEARNING

the cognitive processing activities students opt to use

(Resp10) memorizing stuff … it misses the point, because if you are going to swot like that just to pass a test, what will that help? … But I mean, *sometimes your time is just too little and you must just go and swot the stuff parrot-fashion*, but I don't like doing it like that at all.

(Resp4) If I go back to to second year [names module]. There was those true false questions … *You didn't study like in facts in the way that you would if you had to give a paragraph. You'd study it to understand it, because that's basically the true false questions were testing your understanding*. ... if it's going to be multiple choice questions ... this and this is a complication of that, you only need to recognize it. You don't have to think it out yourself. ... if you've seen it before and you understand how it makes sense as a complication, you're not going to study it

C2. REGULATION OF LEARNING

C2a(i). allocation of effort:

whether students choose to allocate effort to studies rather than to other aspects of their lives

(Resp8) the positive effect of [assessment] for me is that *it forces me to learn* and sometimes when one's self-discipline is just not that good, you are still going to have to learn and *you know the assessment is coming* and it is… it is… in that respect, *it is an incentive to learn*.

(Resp10) I don't really see tests as a way to get points, really, *I just see the tests… in general, I just see motivation to go and learn the work.* I don't know, I can't know if I could honestly say that I would have gone and swotted the work if there now wasn't a test that was going to assess it, but, you know, I like to think that I would, but I don't know.

(Resp13) I learn rather well under stress … I swot in a more disciplined way … *I will say no to [other] stuff and so more, well, easily then… if I know I am under pressure, and I have little time*

C2a(ii). quantity of effort:
the amount and intensity of effort allocated to studies

(Resp13) it depends how close we are to tests, if it is very… *if it is, half, two weeks, I will start swotting immediately [I get home], an hour or so, until say six o'clock, and then relax until about whatever... eight o'clock and then study further.* Otherwise I won't if it is, say, three thirty, I won't just study for an hour in the afternoon, then I will first wait for the evening.

(Resp16) I start learning the Sunday, yes, fine, and my focus is on the learning, and that's what my day is about and I don't do anything else, I don't actually go out or anything, but I do much less. *It is like an eventual curve, half, of how much you do per unit of time*, like, in the beginning it's still just like half like this and then it rises until… *before your test you can learn three, four lectures in half an hour before the test, where you can still actually answer questions out of it, while five days ago you would have taken a whole evening to learn it.*

C2a(iii). distribution of effort:
how effort is allocated to studies over time

(Resp2) I think that because we write tests periodically, I swot periodically. … I think *[the block method] has definitely caused me to change from a person who worked continuously to a person who does the minimum for the first three weeks of every month and the maximum in the last week.*

(Resp9) *the workload of a module is going to determine when your stress level is going to kick in, of when you are going to start learning.* So if it is a module with a high workload, then I will start earlier in the second week. Say Monday. If I know, 'Aah, there is still lots of time, I don't actually have to do that much yet' then I might only start learning the Thursday or the Friday.

C2b. choice of resources:
the resources students opt to utilize when studying

(Resp1) … with [names module] that we did last year. They kind of said to us we don't want you to study from the lecture notes we want you to study from [the] prescribed textbook. … but *if we had studied from [names textbook], we wouldn't have passed the test.* So, you know, they've got their questions in their notes and that's basically what you have to study to get through.

(Resp10) *If there is really a bad lectures or study material that one had, before I [will use journals, the internet or PubMed].* … it would have been very nice, but I mean there is plain and simply not enough time. I know [names module] last year… I mean our notes were unbelievably poor and out textbook was even worse. And I was forced… *I looked at what they put up as other resources and then at the end of it all, I pulled a couple of [continuing medical education] articles.* And is was very nice. … but it was the only time that I ever used the internet or such places in my course, yes… … You just don't have time. It would have been very nice, but I just don't get the time to do it.

(Resp13) *[When I sit with a pack of notes] I at least have the textbook next to me too*, so if I, say now with one or other disease… I'll just scan quickly, quickly, quickly in the book if there is something more than is in the notes. … the PowerPoint is often just the points, the book is at least a little more a story to really gain some insight as to what it all is… *so I will quickly read through that before I read just the PowerPoint just like that. But that is at the beginning then there is still lots of time, and if there is less time, then I just go through the PowerPoint.* … Yes, here around the second week… about four days before the test, I won't do that any more.

C2c. choice of content:
having selected what resources to utilize, students make a second set of decisions

about what content to study from those resources

(Resp7) what I will do is I will see a question paper, I browse through the whole paper, the whole paper is just five to ten point "listing" questions. They're not paragraph or insight or case-studies… Or the case studies are actually just again just to get the little lists out of you in another way… And so I will concentrate on that. *If I page through that work and here is some or other description of a thing, I'll maybe skip over it, but you'll stop at a five or ten point list.* Yes, then one just swots like that, you know, that is what will get you through.

C2d. monitoring & adjustment strategies:

passive or active (e.g., self-testing) checking that learning is proceeding according to plan; and adjusting learning (e.g., activities; contents; or goals) based on monitoring

(Resp9) assessment also has an impact on yourself because it can also allow you … *it also lets you see how you are progressing, whether you are progressing well or whether you are progressing with the class or whether you are behind, whether you must put in more to keep up with the class or whether you are above the class or whether you can relax a bit*

(Resp12) closer to the time I would sort of have glimpse of a question paper if there's any. Yes, I think I usually use a question paper more than the actual outcomes. … sometimes the outcomes are a pain to try and understand what exactly they are. And then so I use the question paper exactly as I should have been using the outcome, to try and understand what is more important. … What I usually do, I, I, I usually look at three question papers from different years. And I just look at it, just look at the questions, and then I just try to answer them, *after having read a certain section. I just try and answer the question paper. And if I am not able to answer on my own, I go back to the section and I, I help myself from the notes to answer the question.* … I use the question paper to test, to test what, what, what, what I have already learned, and also to see what's more important.

C2e. persistence with learning:

whether students persist in allocating time and effort to learning in the face of distractions or fatigue

(Resp4) sometimes the modules do that. So it's a four weeks module and you have [a test] two weeks into the modules and then a test at the end. Some of them do that. … I don't like it. Because, obviously *you get to the stage of like, when you feel you've worked hard on your last clinical block and you feel like relaxing, socialising,* then you see you've got a test. The first week of the module: *not a good way to start. But I mean it definitely makes you start earlier.*

References

1. Cilliers FJ, Schuwirth LWT, Adendorff HJ, Herman N, van der Vleuten CPM: **The mechanism of impact of summative assessment on medical students' learning**. *Advances in Health Sciences Education* 2010, **15**(5):695-715.
2. Cilliers FJ, Schuwirth LWT, Herman N, Adendorff H, van der Vleuten CPM: **A Model of the Pre-assessment Learning Effects of Summative Assessment in Medical Education**. *Advances in Health Sciences Education* 2012, **17**(1):39-53.
